# Renal Function Abnormalities among Patients with Acute Stroke at a Tertiary Hospital in Ethiopia

**DOI:** 10.4314/ejhs.v32i6.15

**Published:** 2022-11

**Authors:** Lissane Seifu, Yonas Gashaye, Melaku Taye

**Affiliations:** 1 Department of Internal Medicine, College of Health Science, Addis Ababa University, Addis Ababa, Ethiopia; 2 Department of Public Health, Santé Medical College, Addis Ababa, Ethiopia

**Keywords:** Acute stroke, Renal dysfunction, Mortality, Ethiopia

## Abstract

**Background:**

Stroke is the leading cause of neurological disability and the second commonest cause of death globally. Despite the fact that renal dysfunction is a common comorbidity of stroke, there is no data on the prevalence of renal dysfunction among patients with acute stroke in Ethiopia. The aim of this study was to determine the magnitude of renal dysfunction, factors associated with renal dysfunction and risk of in-hospital mortality.

**Methods:**

A hospital-based cross-sectional study was conducted in Yekatit 12 Hospital Medical College among consecutive 192 patients, who were admitted with acute stroke from September 2020 to September 2021. Data were collected using a structured questionnaire after pilot survey was done. A Multivariate binary logistic regression analysis was fitted to identify determinants of renal function abnormalities. Renal dysfunction was defined as serum creatinine >1.2mg/dl.

**Result:**

The mean age (SD) of study participants was 62.2 (15.9) years. Hundred-one (52.6%) participants were males. Thirty-four (17.7%) of the participants had renal dysfunction. Among patients with renal dysfunction, more than half of them were ≥70 years old and two-thirds were males. Male gender and hypertension increased the risk of renal dysfunction among hospitalized stroke patients. The mortality rate was higher in stroke patients with renal dysfunction (35.3%) as compared with patients having normal renal function (15.2%), but it was not a statistically significant.

**Conclusion:**

Renal dysfunction was a frequent comorbidity among acute stroke patients who were hospitalized. Male gender and hypertension were statistically significant predictors of renal dysfunction. Mortality rate was higher in stroke patients with renal dysfunction, but not a statistically significant predictor of post stroke in-hospital mortality.

## Introduction

Stroke is the leading cause of neurological disability and the second commonest cause of death in the world (1). Chronic kidney disease (CKD) is frequently associated with cardiovascular diseases; it is considered a cardiovascular risk equivalent. Patients with CKD are more likely to die of cardiovascular diseases (CVD) than to eventually develop renal failure requiring renal replacement therapy (2). Conversely, diagnosis of CKD is higher among patients with cardiovascular diseases than in the general population (3).

CKD is associated with a significantly increased risk of cerebrovascular disease. Even subtle kidney dysfunction, as suggested by albuminuria, increases stroke risk. Stage 3 CKD with microalbuminuria increased stroke risk 1.5 to 2 fold (3). Dialysis patients have six times increased risk of stroke. Stroke accounts for 3% of deaths in end-stage renal disease (ESRD). Stroke in CKD could be ischemic, hemorrhagic, or both; Infarction strokes are more prevalent than hemorrhages. Risk factor reduction (primary and secondary prevention therapies) is the mainstay of therapy (3,3).

Acute kidney injury (AKI) complicating acute stroke is common with an estimated incidence of 8–21% (5). Largely, stroke patients with severe neurological deficits, cardiac abnormalities such as heart failure, atrial fibrillation and ischemic heart disease, hyperglycemia, hypertension, low estimated Glomerular Filtration Rate (eGFR), or advanced age were more susceptible to developing AKI (5–8). It is associated with increased adverse stroke outcomes and mortality, with increased severity of AKI paralleling death risk. Therefore, acute management of kidney dysfunction after stroke may be important to improve post stroke outcome and decrease mortality rates (5–8).

The traditional risk factors for cerebro-renal link are shared with the general population but are more prevalent in patients with kidney diseases. These include hypertension, dyslipidemia, hypervolemia, sympathetic over activity and hyperhomocytinemia. In addition, novel factors (CKD-related) are also postulated to further increase the risk of stroke. These are generalized inflammation, anemia, hypophosphatemia, hyperparathyroidism, increased fibroblast growth factor 23 (FGF-23) and sleep apnea (8).

There is a dearth of information about the magnitude of renal dysfunction in patients with acute stroke in Ethiopia. We, therefore, conducted a cross-sectional study to assess the magnitude of renal dysfunction, factors associated with renal dysfunction and in-hospital mortality in patients with acute stroke.

## Materials and Methods

**Study period, area and design**: An institution-based cross-sectional study was conducted in Yekatit 12 Hospital Medical College, Addis Ababa, Ethiopia. This hospital, with a total bed capacity of 265, provides inpatient services for more than 22000 patients each year. The study was conducted from September 2020 to September 2021.

**Study population**: The study population was patients with acute stroke hospitalized in Yekatit 12 Hospital Medical College during the study period.

**Sample size, sampling technique and exclusion criteria**: Non-probability convenient sampling method was used to enroll every consecutive patient hospitalized with acute stroke and had serum creatinine measurement within 24 hours of admission. Patients without renal function assessment with in the first 24 hours were excluded.

**Data collection tools and procedures**: Data were collected by trained personnel from the patients' medical records from admission to discharge or death using a structured data collection tool which was developed in English. Socio-demographic characteristics, family history of kidney disease and lifestyle behaviors, comorbidities, clinical profiles, laboratory values and in-hospital outcomes were abstracted from medical records. Vital signs, Glasgow coma scale (GCS) and random blood sugar (RBS) were taken from a record of emergency triage evaluation document. Among laboratory results documented within the first 24 hours of admission, the first records were taken as laboratory profiles.

**Operational definitions**: All comorbidities were defined as present if documented in the medical records by the treating physician. Since the courses of the renal functions of the patients before three months were unknown, we used serum creatinine rather than eGFR for the operational definition of impaired renal function. Recognized renal dysfunction was defined as having serum creatinine of >1.2 mg/dl (10). Albuminuria was determined using rapid test strips and was defined as a dipstick of ≥1+.

**Data quality assurance, processing and management**: To assure the quality of data, pilot survey was conducted and a structured data collection tool was prepared and used for data collection. During the data collection procedures, all the collected data were reviewed and checked for their completeness everyday by the supervisor and weekly by the principal investigator. The collected data were validated and exported to the statistical package for social science (SPSS) 26 for analysis. Descriptive statistics included mean with SD for continuous variables, while frequency and percentage tables were used for categorical data. Simple cross-tabulation and binary logistic regression analysis were used to study the association of independent variables with the renal dysfunction and in-hospital outcome. Variables with P-value of <0.25 in the bivariate analysis were entered together into multivariable analysis to control for confounders. Then, variables with p-value of <0.05 in multivariable analysis were considered as statistically significant and AOR with 95% CI was estimated to measure the strength of the associations. The results were presented by using text and tables.

**Ethical considerations**: The confidentiality of all participants' records and documents was respected. The names of the study subjects or hospital identification numbers were not listed in the questionnaire; rather, codes were used and more sensitive information was kept private. Consent was not taken since only medical records were used. Ethical approval was obtained from the institutional review board of Santé Medical College and permission was granted from Yekatit 12 Hospital Medical College. Data were analyzed anonymously.

## Results

Two hundred fifty-six patients, with the diagnosis of acute stroke, were admitted during the study period, but sixty-four patients serum creatinine level were not determined within 24 hours of admission and, hence, were excluded from the study. A total of 192 patients participated in this study with the mean age (SD) of 62.2 (15.9) years. Seventy nine (41.1%) were in the ages ≥70 years and 101 (52.6%) were males. Nearly two thirds (64.6%) of patients presented with ischemic stroke. Sixty-three (50.8%) of patients who had ischemic stroke were females and 40 (58.8%) of hemorrhagic stroke patients were males.

The minimum and maximum values of serum creatinine of the study participants were 0.3 and 6.6 mg/dl, respectively, with mean (SD) of 0.9 (0.5). Thirty-four (17.7%) of the study participants had renal dysfunction. Patients with renal dysfunction were ≥70 years old constituting more than half (52.9%) and were predominantly males (67.6%).

Hypertension was the most frequent cardiovascular comorbidity in 117 (60.9%) of patients and there was a higher prevalence of hypertension in patients with renal dysfunction (76.5%) as compared to patients with normal renal function. History of diabetes mellitus (DM) and previous transient ischemic stroke (TIA)/stroke were more common in patients with renal dysfunction as well. Among patients with renal dysfunction, 47.1% and 64.7% had blood pressure (BP) ≥160/100 mmHg and decreased mentation, GCS <15, respectively, at admission.

Table 1 and Table 2 summarize the sociodemographic and clinical features, and laboratory profiles respectively.

**Table 1:** Sociodemographic and clinical profiles of stroke patients at admission based on their renal function group at Yekatit 12 Hospital Medical College, Addis Ababa, Ethiopia, 2020/21

Clinical profiles		Renal dysfunction (n=34)		Normal renal function (n=158)	
		
		n	%	n	%
Age in years	≥70	18	52.9	61	38.6
	<70	16	47.1	97	61.4
Sex	Male	23	67.6	78	49.4
	Female	11	32.4	80	50.6
History of HTN	Yes	26	76.5	91	57.6
	No	8	23.5	67	42.4
History of DM	Yes	7	20.6	22	13.9
	No	27	79.4	136	86.1
History of previous TIA/stroke	Yes	6	17.6	13	8.2
	No	28	82.4	145	91.8
History of cardiac disease	Yes	2	5.9	15	9.5
	No	32	94.1	146	90.5
BP in mmHg	≥160/100	16	47.1	69	43.7
	<160/100	18	52.9	89	56.3
PR in beats per minute	≥100	11	32.4	40	25.3
	<100	23	67.6	118	74.7
GCS	<15	22	64.7	86	54.4
	15	12	35.3	72	45.6
Stroke type	Ischemic	21	61.8	103	65.2
	Hemorrhagic	13	38.2	55	34.8

DM – diabetes mellitus, TIA – Transient ischemic stroke, HTN – Hypertension, GCS – Glasgow coma scale, mmHg – millimeter mercury.

**Table 2:** Laboratory test profiles of stroke patients at admission based on their renal function group at Yekatit 12 Hospital Medical College, Addis Ababa, Ethiopia, 2020/2.

Lab profile		Renal dysfunction		Normal renal function	
		
		N	%	n	%
RBS in mg/dl (n=191)	≥200	7	20.6	15	9.6
	<200	27	79.4	142	90.4
Urine dipstick (n=159)	≥+1	31	100.0	15	11.7
	<+1	0	0	113	88.3
Total cholesterol in mg/dl	≥200	4	22.2	20	20.8
(n=114)	<200	14	77.8	76	79.2
HDL in mg/dl (n=99)	<35	3	17.6	14	17.1
	≥35	14	82.4	68	82.9
LDL in mg/dl (89)	≥130	4	28.6	24	32.0
	<130	10	71.4	51	68.0
Triglyceride in mg/dl (n=107)	≥200	1	5.9	7	7.8
	<200	16	94.1	83	92.2
Sodium (Na) (n=153)	<135	9	34.6	45	35.4
	≥135	17	65.4	82	64.6
Potassium (K) (n=151)	<3.3	3	12.0	15	11.9
	≥3.3	22	88.0	111	88.1
Chloride (Cl) (149)	<98	10	40.0	48	38.7
	≥98	15	60.0	76	61.3

Lab – Laboratory; RBS – Random blood sugar, HDL – High-density lipoprotein, LDL – Low-density lipoprotein

In the full model of multivariate regression analysis, the following parameters were included: age, sex, HTN, DM, previous TIA/stroke, history of cardiac disease, admission BP, GCS, stroke type, admission RBS, total cholesterol, HDL, LDL and triglyceride. Stepwise logistic regression analysis was performed including variables with p<0.25 in the full model. Multivariate analysis showed that the independent predictor of renal dysfunction in patients with acute stroke were male sex (AOR = 2.43, 95% CI: 1.06, 5.55, p=0.036) and hypertension (AOR = 2.57, 95% CI: 1.05, 6.29, p=0.039) (Table 3).

**Table 3:** Subgroup analysis of factors associated with renal dysfunction among stroke patients at Yekatit 12 Hospital Medical College, Addis Ababa, Ethiopia, 2020/21

Variables	Category	RD	Normal RF	COR (95%,CI)	P-value	AOR (95%,CI)	P-value
Age (years)	≥70	52.9	38.6	1.79(0.85,3.77)	0.126	1.89(0.85,4.19)	0.117
Sex	Male	67.6	49.4	2.14(0.98,4.69)	0.056	2.43(1.06,5.55)	0.036
HTN	Yes	76.5	57.6	2.39(1.02,5.62)	0.045	2.57(1.05,6.29)	0.039
Previous TIA/stroke	Yes	17.6	8.2	2.61(0.90,7.53)	0.076	1.89(0.62,5.74)	0.26
RBS(mg/dl)	≥200	20.6	9.6	2.45(0.91,6.58)	0.075	2.08(0.75,5.86)	0.167

RD = Renal dysfunction, RF = Renal function, COR= Crude odds ratio; CI = Confidence interval; AOR = Adjusted odds ratio; TIA =Transient ischemic attack., RBS = Random blood sugar, HTN = Hypertension.

During the period of hospitalization, 36 (18.75%) of the study participants were dead, with one third (33.3%) of them having had renal dysfunction. The proportions of mortality were significantly different between the two groups, with disproportionately higher rate in patients with renal dysfunction. In the full model of multivariate regression analysis for in-hospital mortality, age, sex, HTN, DM, previous TIA/stroke, history of cardiac disease, admission BP, GCS, stroke type, admission RBS, total cholesterol, HDL, LDL, triglyceride and renal dysfunction were considered. Stepwise logistic regression analysis was performed including variables with p<0.25. Multivariate analysis showed that renal dysfunction was not a statistically significant risk factor of post stroke in-hospital mortality (AOR=1.45, 95% CI, 0.21, 9.91, p=0.702). But, the model was influenced by HDL<35 mg/dl (AOR = 5.86, 95% CI, 1.35, 25.52, p=0.019) (Table 4).

**Table 4:** Multivariate logistic regression analysis of factors associated with death among stroke patients at Yekatit 12 Hospital Medical College, Addis Ababa, Ethiopia, 2020/21

Variables	Categories	In hospital outcome		COR (95%,CI)	AOR (95%,CI)	P-value
					
		Died (%)	Alive (%)			
Age in years	≥70	55.6	37.8	2.05(0.99,4.28)	4.30(0.97,19.11)	0.055
Cardiac disease	Yes	19.4	6.4	3.52(1.24,10.02)	2.60(0.37,18.04)	0.33
GCS	<15	86.1	49.4	6.36(2.35,17.21)	2.73(0.60,12.47)	0.196
RBS in mg/dl	≥200	5.6	12.9	0.40(0.09,1.78)	0.39(0.03,5.12)	0.47
Renal dysfunction	Yes	33.3	14.1	3.04(1.33,6.96)	1.45(0.21,9.91)	0.70
HDL in mg/dl	<35	45.5	13.6	5.28(1.39,20.03)	5.86(1.35,25.52)	0.019

GCS = Glasgow coma scale, RBS = Random blood sugar, HDL = High-density lipoprotein.

## Discussion

To our knowledge, no previous investigation on renal function abnormalities among stroke patients has been undertaken in Ethiopia. This study assessed the magnitude of renal dysfunction in patients hospitalized with acute stroke and its association with in-hospital stroke mortality.

The magnitude of renal dysfunction among hospitalized acute stroke patients was 17.7% which was consistent with a study done in Israeli based on the prospective National Acute Stroke Israeli (NASIS) registry in 2016 (10), and a prospective study conducted in 352 stroke patients in Poland (10). According to a cohort study of 1350 hospitalized first-ever stroke patients conducted in Athens, Greece, over a 10-year period, 28.1% of acute stroke patients had moderate or severe renal impairment (12), which was higher than our finding. Small sample size and that it only included patients with recognized renal dysfunction could have underestimated the magnitude of renal dysfunction in this study. A prospective cohort study conducted among 52 patients admitted in intensive care unit in Nepal from 2014 to 2015 showed that 48.1% had renal impairment (13). This outlier discrepancy could be due to significant overestimation of GFR, because direct measurement of Cr clearance to determine renal function is imprecise in critically ill patients due to the increased secretion of Cr in the renal tubules (13).

This study showed that more than two-thirds of stroke patients with renal dysfunction were males which is slightly higher than a study report of Israel in 2016 (10). In this study, being male was also a statistically significant risk factor which increased the risk of renal dysfunction by a factor of 2.43 (AOR = 2.43, 95% CI: 1.06, 5.55, p=0.036) after adjusted for confounding factors (age, HTN, previous TIA/stroke and RBS). A study conducted in Greece among 335 stroke patients with renal dysfunction reported that male gender had a statistically significant association with renal impairment (p=0.000) (12).

We also found that hypertension was highly prevalent in acute stroke patients with renal dysfunction. This rate is consistent with the finding of a study conducted in Greece in 2008 (77.6%) (12). In contrast, a cohort study in Israel found that the prevalence of hypertension in stroke patients with renal dysfunction was 88.7% (10). This difference may be due to the fact that many potential risk factors of hypertension (like smoking, dietary habits, ageing, not being physically active, urbanization) are more common in developed countries as compared with developing countries like Ethiopia. A multivariate analysis also showed that hypertension was a statistically significant predictor of renal dysfunction among acute stroke patients with renal dysfunction (AOR = 2.57, 95% CI: 1.05, 6.29, p=0.039). This finding is supported by a study in Greece conducted among 335 stroke patients with renal dysfunction (p=0.000) (12).

Studies suggested that renal impairment was an independent predictor for cardiovascular accident outcome and all-cause mortality in high risk population including the elderly (2, 15). Sweileh et al., identified three predictors of inhospital mortality in stroke patients: creatinine clearance (p = 0.004), number of post-stroke complications (p = 0.001), and type of stroke (p = 0.034), (16). Other study also showed that serum creatinine of >1.3mg/dL was associated with increased risk of cardiovascular mortality (17). Our study showed that the mortality rate was disproportionately higher in stroke patients with renal dysfunction as compared to patients having normal renal function, but it was not a statistically significant risk factor of post stroke in-hospital mortality (AOR=1.45, 95% CI, 0.21, 9.91, p=0.702). Factors associated with impaired renal function that may contribute to the adverse outcome of patients with stroke include: insulin resistance, oxidative stress, inflammation, endothelial dysfunction, vascular calcification, and increased plasma levels of fibrinogen and homocysteine (10).

The limitations of this study include: First, its a small sample size and the fact that it was institution based, which may not represent the general population. Secondly, varying laboratory standards of renal function measurements could have underestimated/ overestimate the magnitude of renal dysfunction in our study. Third, we were unable to calculate eGFR, hence, serum Cr was determined only at a single point in time, on admission, and the patient was in acute medical condition. However, being the first study conducted in Ethiopia on renal function abnormalities among stroke patients is the strength of this study.

Renal dysfunction was a frequent comorbidity in patients hospitalized with acute stroke. Male gender and hypertension were the significant predictors of renal dysfunction among stroke patients. Although patients with renal dysfunction had higher mortality rate, it had no statistically significant association with in-hospital post stroke mortality.
